# Assessment of thermal distribution through an inclined radiative-convective porous fin of concave profile using generalized residual power series method (GRPSM)

**DOI:** 10.1038/s41598-022-15396-z

**Published:** 2022-08-02

**Authors:** R. S. Varun Kumar, G. Sowmya, M. C. Jayaprakash, B. C. Prasannakumara, M. Ijaz Khan, Kamel Guedri, Poom Kumam, Kanokwan Sitthithakerngkiet, Ahmed M. Galal

**Affiliations:** 1 Department of Mathematics, Davangere University, Davangere, Karnataka 577002 India; 2 Department of Mathematics, M S Ramaiah Institute of Technology, Bangalore, Karnataka 560054 India; 3 Department of Information Technology, University of Technology and Applied Sciences, Al Mussanah, Sultanate of Oman; 4 Department of Mathematics and Statistics, Riphah International University, I-14, Islamabad, 44000 Pakistan; 5 Nonlinear Analysis and Applied Mathematics (NAAM)-Research Group, Department of Mathematics, Faculty of Sciences, King Abdulaziz University, P.O. Box 80203, Jeddah, 21589 Saudi Arabia; 6 Mechanical Engineering Department, College of Engineering and Islamic Architecture, Umm Al-Qura University, P.O. Box 5555, Makkah, 21955 Saudi Arabia; 7 Center of Excellence in Theoretical and Computational Science (TaCS-CoE), KMUTT Fixed Point Research Laboratory, Room SCL 802 Fixed Point Laboratory, Science Laboratory Building, Departments of Mathematics, Faculty of Science, King Mongkut’s University of Technology Thonburi (KMUTT), 126 Pracha-Uthit Road, Bang Mod, Thung Khru, Bangkok, 10140 Thailand; 8 Department of Medical Research, China Medical University Hospital, China Medical University, Taichung, 40402 Taiwan; 9 Intelligent and Nonlinear Dynamic Innovations Research Center, Department of Mathematics, Faculty of Applied Science, King Mongkut’s University of Technology North Bangkok (KMUTNB), 1518, Wongsawang, Bangsue, Bangkok, 10800 Thailand; 10 Mechanical Engineering Department, College of Engineering, Prince Sattam Bin Abdulaziz University, Wadi ad-Dawasir, 11991 Saudi Arabia; 11 Production Engineering and Mechanical Design Department, Faculty of Engineering, Mansoura University, P.O. 35516, Mansoura, Egypt

**Keywords:** Engineering, Mathematics and computing

## Abstract

The thermal distribution in a convective-radiative concave porous fin appended to an inclined surface has been examined in this research. The equation governing the temperature and heat variation in fin with internal heat generation is transformed using non-dimensional variables, and the resulting partial differential equation (PDE) is tackled using an analytical scheme, generalized residual power series method (GRPSM). Moreover, a graphical discussion is provided to examine the consequence of diverse non-dimensional variables including the parameters of convection-conduction, ambient temperature, radiation, heat generation, and porosity effect on the thermal field of the fin. Also, a graph is plotted to analyze the variations in unsteady temperature gradient using the finite difference method (FDM) and generalized residual power series method (GRPSM). The major result of this investigation unveils that as the convection-conduction parameter scale upsurges, the distribution of temperature in the fin diminishes. For the heat-generating parameter, the thermal distribution inside the fin increases.

## Introduction

Heat transfer is the transmission of energy induced by temperature variations and if two systems in contact have different temperatures, heat transfer occurs until thermal equilibrium is reached. The innovation of effective heat transfer liquids with elevated thermal conductivity and heat transfer coefficient is required to improve the efficiency of the heat transmission process and to reduce the cost and size of the relevant modules and devices. The suspension of tiny solid particulate in liquids is an effective method of increasing the thermal conductivity of liquids and thereby enhances the heat transference phenomenon. Using these kinds of liquids, several investigators explored the features of heat transferal^1–7^. On the other side, heat transfer is enhanced using the extended surface. Excessive heat is produced in machine parts in several industrial applications, which can lead to a variety of material flaws. Heat transfer through the extended surface of the apparatus is one strategy for avoiding material damage. A fin is an extended surface that is utilized to augment the rate of heat transference from the primary surface to the environment. It has extensive technological applications, namely air-cooled craft engines, compressors, nuclear reactors, heat exchangers, refrigeration, electrical and electronic apparatuses, and so on. Meanwhile, porous material fins have considerable advantages over conventional fins, and their research is one of the most comprehensive topics in the field of mass and energy transference. In the heat transferal scrutiny of permeable material fins, energy and mass transference of both solid and fluid media must be taken into account. Several analyses have been performed to explore efficient and productive methods of heat transferring through permeable finned surfaces. Ndlovu and Moitsheki^8^ discussed the one-dimensional heat transport and thermal aspects in a moveable porous straight fin of the uniform area of cross-section. With the impact of radiative, magnetic, and convective mechanisms, Madhura et al.^9^ depicted the features of the thermal field of a permeable longitudinal fin. The sinc collocation method was executed to study the thermal behavior of permeable fin by Nabati et al.^10^ under the influence of magnetic force. With the implementation of analytical procedures, Kundu and Yook^11^ determined the analytical approximation of the porous fin and thus investigated the heat transfer features of the considered fin. Considering the local thermal non-equilibrium model, Buonomo et al.^12^ researched the energy transfer aspects of a permeable rectangular extended surface. Implementing the spectral collocation method, Kumar et al.^13^ described the temperature and energy variation in a permeable trapezoidal extended surface with radiation phenomenon.

Fins with a non-uniform cross-sectional area, which contribute to a lighter structure, are recommended in airborne and space application fields over heavier rectangular-shaped fins, despite the fact that such lighter fin constructions are more complicated and costlier to produce. Aziz and Fang^14^ expounded on the thermal variations inside the straight fin of variable thickness. Further heat transfer aspects are discussed by considering various fin profiles namely trapezoidal, rectangular and concave. Using the DTM approach, Torabi et al.^15^ debriefed the thermal performance of radiative-convective concave profiled fin. The heat transference features of the concave parabolic extended surface were discussed by Kang^16^. Recently, Wang et al.^17^ employed the technique of DTM to examine the aspects of energy dissipation through a permeable fin of the inclined surface. The thermal performance and heat flow in a dovetail fin with internal heat production were probed by Goud et al.^18^. In the presence of convection and radiation, Jagadeesha et al.^19^ expounded on the thermal performance of a fully wetted semi-spherical fin using a non-Fourier heat conduction model. Several researchers have worked on solving ordinary differential equations using various techniques, including the differential transform method^20–24^, sinc collocation approach^10^, finite difference method^25^, spectral collocation method^26,27^, least square method^28^ and among others. Several of these methodologies are computationally complex because they are trial-and-error in nature or involve complicated symbolic calculations. The residual power series method (RPSM) is one of the analytical techniques which is broadly utilized to achieve an approximate solution because it does not necessitate any restrictive assumptions or linearization. This method can be employed effectively to the given problems and it is easier to achieve accurate approximate solutions without more complications. The RPSM is an innovative approach for procuring analytical Taylor series solutions for linear and nonlinear differential equations. As a result of applying the residual error notion, a series solution, as well as a truncated series solution, can be obtained. Arqub et al.^29,30^ applied the RPSM for solving the initial value problems. Using RPSM, Syam^31^ discussed the solution of the fractional-order Fredholm integrodifferential equation. Az-Zo’bi^32^ applied RPSM to analyze the numerical solution of the time-dependent motion of the van der Waals gas model. Refs.^33–38^ highlights the importance of fluid flow regarding various assumption via different geometries.

The majority of the studies were focused on analyzing the one-dimensional thermal distribution of the porous straight fin or fin with a tapered profile, as evidenced by the above investigations. Further numerical and analytical solutions are provided in other cases. Also, there are no detailed analyses with analytical solutions on the unsteady thermal distribution through a tapered inclined fin. Thus, the principal objective of this analysis is to scrutinize the unsteady temperature variance through an inclined concave porous fin with internal heating. Moreover, the temperature profile of the fin has been solved analytically using the generalized residual power series method (GRPSM).

## Mathematical formulation

**Figure 1:**
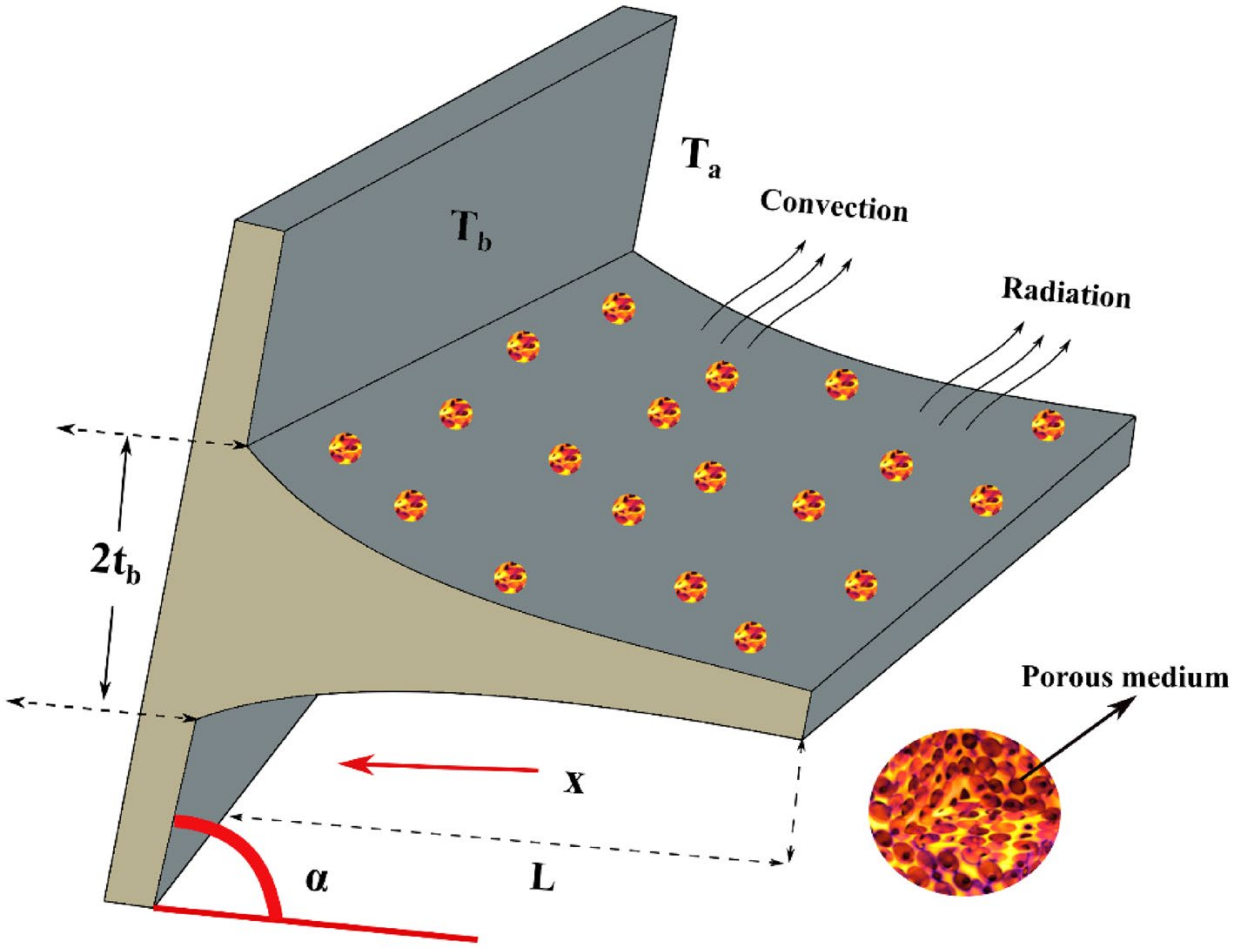
Physical depiction of an inclined concave parabolic fin.

The unsteady thermal performance of permeable concave fin with convection and radiation is studied. $L$ and $W$ are the length and width of the permeable concave fin with base thickness $t_{b}$. The dissipation of heat to the ambient environment occurs due to the effect of radiation and convection at $T_{a}$. The fin which is attached to an inclined surface at an angle $\alpha$ is considered in this analysis, as demonstrated in Fig. 1. It is assumed that the porous medium is saturated with a single phase, homogeneous, and isotropic fluid. Darcy's model characterizes the interaction of a porous medium and fluid media. Another implication involves that the fin's thickness is small in comparison to its length. The temperature inside the fin is also presumed to vary only in the x-direction, with the temperature variation in the y-direction being small enough to ignore.

The transient energy transmission with the above-mentioned assumption is stated by the following governing equation (Torabi et al.^15^, and Ma et al.^39^):

1 $$\begin{aligned} \rho c_{p} t_{b} \frac{\partial T}{{\partial \tau }} & = \frac{\partial }{\partial x}\left[ {\left\{ {k_{f} \phi + k_{s} \left( {1 - \phi } \right)} \right\}t(x)\frac{\partial T}{{\partial x}}} \right] - \left( {1 - \phi } \right)h^{*} \left( {T - T_{a} } \right) + \left( {1 - \phi } \right)t(x)q^{*} (T) \\ & \quad - \frac{{\rho_{f} c_{p} gK\beta_{f} \sin \left( \alpha \right)}}{{\,\nu_{f} }}\left( {T - T_{a} } \right)^{2} - \sigma \varepsilon^{*} \left( {T^{4} - T_{a}^{4} } \right), \\ \end{aligned}$$

where $h^{*}$ is the convective heat transfer coefficient, $q^{*} (T)$ signifies internal heat generation, $\rho$ is the density, $\sigma$ indicates Stefan–Boltzmann constant, $g$ symbolizes acceleration due to gravity, $K$ is the permeability, $c_{p}$ represents specific heat, and $t(x) = \left[ {\Lambda \left( {\left( \frac{x}{L} \right)^{2} - 1} \right) + t_{b} } \right]$ is the local semi-fin thicknesses.

The internal heat generation is presumed to be a function of temperature:

2 $$q^{*} (T) = q_{a} \left[ {1 + \xi \left( {T - T_{a} } \right)} \right],$$

where, $q_{a}$ denotes heat generation at ambient temperature, and $\xi$ is the heat generation parameter. As this analysis considers a finite-length fin with an insulated tip, there will be no heat flow through the tip of the fin. Thus, the relevant boundary conditions (BCs) for Eq. (1) are:

3 $$\begin{aligned} T\left( {x,0} \right) & = T_{a} , \\ T\left( {L,\tau } \right) & = T_{b} , \\ \frac{\partial T}{{\partial x}}_{x = 0} & = 0. \\ \end{aligned}$$

The appropriate non-dimensional terms involved in this study are:

4 $$\begin{gathered} \tau^{*} = \frac{{k_{s} \tau }}{{\rho c_{p} L^{2} }},\quad k_{r} = \frac{{k_{f} }}{{k_{s} }},\quad \Theta = \frac{T}{{T_{b} }},\quad X = \frac{x}{L},\quad C = \frac{\Lambda }{{t_{b} }},\quad Nc = \frac{{h^{*} \left( L \right)^{2} }}{{k_{s} t_{b} }} \hfill \\ \Theta_{a} = \frac{{T_{a} }}{{T_{b} }},\quad Nr = \frac{{\sigma \varepsilon^{*} T_{b}^{3} \left( L \right)^{2} }}{{k_{s} t_{b} }},\quad S_{H} = \frac{{\rho_{f} c_{p} gK\beta_{f} \left( L \right)^{2} T_{b} }}{{\nu_{f} k_{S} t_{b} }}, \hfill \\ Q = \frac{{q_{a} \left( L \right)^{2} }}{{k_{s} T_{b} }},\quad \gamma = T_{b} \xi . \hfill \\ \end{gathered}$$

Equation (1) and BCs are transformed to a non-dimensional form using Eq. (2) and Eq. (4) to yield,

5 $$\begin{aligned} \frac{\partial \Theta }{{\partial \tau^{*} }} & = \frac{\partial }{\partial X}\left[ {\left( {k_{r} \,\varphi + \left( {1 - \phi } \right)} \right)\left( {C\left( {X^{2} - 1} \right) + 1} \right)\frac{\partial \Theta }{{\partial X}}} \right] - S_{H} \sin \left( \alpha \right)\left( {\Theta - \Theta_{a} } \right)^{2} - \left( {1 - \phi } \right)Nc\left( {\Theta - \Theta_{a} } \right) \\ & \quad - Nr\left( {\Theta^{4} - \Theta_{a}^{4} } \right) + \left( {1 - \phi } \right)\left( {C\left( {X^{2} - 1} \right) + 1} \right)Q\left[ {1 + \gamma \left( {\Theta - \Theta_{a} } \right)} \right]. \\ \end{aligned}$$

The above equation includes radiation-conduction parameter $Nr$ which is directly associated with the surface emissivity, fin taper ratio $C$ that describes the concave tapered profile, convection-conduction parameter $Nc$ is the ratio of convention to conduction, internal heat production parameter $Q$, and porosity parameter $S_{H}$.

Simultaneously with the help of Eq. (4), Eq. (3) yields,

6 $$\begin{aligned} \Theta \left( {X,0} \right) & = \Theta_{a} , \\ \Theta \left( {1,\tau^{*} } \right) & = 1, \\ \left. {\frac{\partial \Theta }{{\partial X}}} \right|_{X = 0} & = 0. \\ \end{aligned}$$

## Fundamentals of GRPSM

Let $F$ be a function of two variables $x$ and $t$ i.e.$F(x,t)$ and consider the following PDE,

7 $$\frac{\partial }{\partial t}F(x,t) + \Omega \,F(x,t) = g(x,t),\,\,\,\,\,\,x \in \Gamma, \,\,\,\,\,\,t \in [0,{\rm Z}],$$

with initial and boundary conditions,

8 $$\begin{gathered} F(x,0) = F_{0} (x),\,\,\,\,\,\,\,x \in \Gamma \hfill \\ F(x,t) = h(x,t),\,\,\,\,\,\,\,x \in \partial \Gamma, \,\,\,\,\,\,\,\,t \in {\rm Z}, \hfill \\ \end{gathered}$$

where, $\Omega$ is a differential operator and $g$ denotes the source term.

Assume that the solution of Eq. (7) has the power series in the form shown below

9 $$F(x,t) = \sum\limits_{i = 0}^{k} {Y_{i} (x)t^{i} ,\quad k = 0,1,2,3, \ldots }$$

Rewriting the Eq. (7) yields

10 $$\frac{\partial }{\partial t}F(x,t) + \Omega F(x,t) - g(x,t) = 0.$$

To evaluate the coefficient functions, $Y_{i} (x),\,\,\,i = 0 \ldots k$, kth residual function is defined as

11 $${\text{Re}} s^{k} (t) = \frac{\partial }{\partial t}\left[ {\left( {Y_{0} (x) + \sum\limits_{i = 1}^{k} {Y_{i} (x)t^{i} } } \right)} \right] + \Omega \sum\limits_{i = 0}^{k} {Y_{i} (x)t^{i} - g(x,t)}.$$

Reiterating this operation to find the *n*th-truncated solution yields

12 $$F\left( {x,t} \right) = Y_{0} (x) + Y_{1} (x)t + Y_{2} (x)t^{2} + Y_{3} (x)t^{3} + \cdots.$$

## Application of GRPSM

Rearrange the Eq. (5) as:

13 $$\begin{aligned} & \frac{\partial \Theta }{{\partial \tau^{*} }} - \frac{\partial }{\partial X}\left[ {\left( {k_{r} \,\varphi + \left( {1 - \phi } \right)} \right)\left( {C\left( {X^{2} - 1} \right) + 1} \right)\frac{\partial \Theta }{{\partial X}}} \right] + S_{H} \sin \left( \alpha \right)\left( {\Theta - \Theta_{a} } \right)^{2} + \left( {1 - \phi } \right)Nc\left( {\Theta - \Theta_{a} } \right) \\ & \quad + Nr\left( {\Theta^{4} - \Theta_{a}^{4} } \right) - \left( {1 - \phi } \right)\left( {C\left( {X^{2} - 1} \right) + 1} \right)Q\left[ {1 + \gamma \left( {\Theta - \Theta_{a} } \right)} \right] = 0. \\ \end{aligned}$$

Let the series solution be in the form

14 $$\Theta \left( {X,\tau^{*} } \right) = \sum\limits_{k = 0}^{m} {\Psi_{k} \left( X \right)\left( {\tau^{*} } \right)^{k} }.$$

Applying the GRPSM to Eq. (13) gives

15 $$\begin{aligned} {\text{Re}} s^{k} \left( {X,\tau^{*} } \right) & = \frac{\partial }{{\partial \tau^{*} }}\left( {\sum\limits_{k = 0}^{m} {\Psi_{k} \left( X \right)\left( {\tau^{*} } \right)^{k} } } \right) - \frac{\partial }{\partial X}\left[ {\left( {k_{r} \,\varphi + \left( {1 - \phi } \right)} \right)\left( {1 + C\left( {X^{2} - 1} \right)} \right)\frac{\partial }{\partial X}\left( {\sum\limits_{k = 0}^{m} {\Psi_{k} \left( X \right)\left( {\tau^{*} } \right)^{k} } } \right)} \right] \\ & \quad + S_{H} \sin \left( \alpha \right)\left( {\left( {\sum\limits_{k = 0}^{m} {\Psi_{k} \left( X \right)\left( {\tau^{*} } \right)^{k} } } \right) - \Theta_{a} } \right)^{2} + \left( {1 - \phi } \right)Nc\left( {\left( {\sum\limits_{k = 0}^{m} {\Psi_{k} \left( X \right)\left( {\tau^{*} } \right)^{k} } } \right) - \Theta_{a} } \right) + Nr\left( {\left( {\sum\limits_{k = 0}^{m} {\Psi_{k} \left( X \right)\left( {\tau^{*} } \right)^{k} } } \right)^{4} - \Theta_{a}^{4} } \right) \\ & \quad - \left( {1 - \phi } \right)\left( {1 + C\left( {X^{2} - 1} \right)} \right)Q\left[ {1 + \gamma \left( {\left( {\sum\limits_{k = 0}^{m} {\Psi_{k} \left( X \right)\left( {\tau^{*} } \right)^{k} } } \right) - \Theta_{a} } \right)} \right]. \\ \end{aligned}$$

To obtain the coefficients $\Psi_{k} \left( X \right),\,\,k = 1,2,3, \ldots ,m$, replace $k$th a truncated series of $\Theta \left( {X,\tau^{*} } \right)$ in Eq. (15), and apply the below derivative formula on ${\text{Re}} s^{k} \left( {X,\tau^{*} } \right)$ (Modanli et al.^40^),

16 $$\left. {\frac{{\partial^{h} }}{{\partial \tau^{*h} }}{\text{Re}} s^{k} \left( {X,\tau^{*} } \right)} \right|_{{\tau^{*} = 0}} = 0,\quad h = 1,2,3, \ldots ,m.$$

For simplificantion, the values for the corresponding parameters are taken as $Nc = 2$, $S_{H} = 0.5$, $Nr = 3$, $\Theta_{a} = 0.2$, $k_{r} = 0.1$, $C = 0.1$, $\phi = 0.1$, $\gamma = 0.2$, $Q = 0.8$ and $\alpha = {\pi \mathord{\left/ {\vphantom {\pi 6}} \right. \kern-\nulldelimiterspace} 6}$. Upon substituting these values and with the use of BCs the coefficients of $\Psi_{k} \left( X \right),\;k = 1,2,3, \ldots ,8$ are determined,

17 $$\Psi_{0} = {\rm M},\quad \Psi_{1} = 1,$$

18 $$\Psi_{3} = \Psi_{5} = \Psi_{7} = 0,$$

19 $$\Psi_{2} = 1.831501832\,{\rm M}^{4} - 0.5963858364 + 0.9587301587\,{\rm M} + 0.1526251526\,{\rm M}^{2}$$

20 $$\begin{aligned} \Psi_{4} & = { }0.06695131846\,{\rm M}^{2} + 1.388721169\,{\rm M}^{4} - 0.07102258678 + 0.08383240328\,{\rm M} \\ & \quad + 0.2795332466\,{\rm M}^{5} - 0.7204230222\,{\rm M}^{3} + 2.236265973\,{\rm M}^{7} \\ \end{aligned}$$

21 $$\begin{aligned} \Psi_{6} & = - 0.01045231395\,{\rm M} + 0.001392072268 - 1.923825243\,{\rm M}^{6} + 0.6257357983\,{\rm M}^{8} \\ & \quad + 3.549628529\,{\rm M}^{10} - 0.8906991152\,{\rm M}^{3} + 0.5878481440\,{\rm M}^{4} + 0.2718739855\,{\rm M}^{2} \\ & \quad + 0.3198549028\,{\rm M}^{5} + 3.321141670\,{\rm M}^{7} \\ \end{aligned}$$

22 $$\begin{aligned} \Psi_{8} & = - 0.05685639053{\rm M} + 0.0004730083722 + 5.751018328{\rm M}^{13} - 4.073041686{\rm M}^{9} \\ & \quad + 1.318685879{\rm M}^{11} - 3.151272005{\rm M}^{6} + 1.069742989{\rm M}^{8} + 7.101219309{\rm M}^{10} \\ & \quad - 0.4787022153{\rm M}^{3} + 0.09124498829{\rm M}^{4} + 0.3033104496{\rm M}^{2} + 1.101500411{\rm M}^{5} \\ & \quad + 2.140222196{\rm M}^{7}. \\ \end{aligned}$$

Express the above coefficients in a truncation series and ${\text{M}} = 0.5937942627$ is the value obtained with the help of BC. Using the achieved $k$th truncated series in Eq. (14), the final series solution of the fin problem is represented as

23 $$\begin{aligned} \Theta \left( {X,\tau^{*} } \right) & = 0.5937942627 + 0.2544108558\,\left( {\tau^{*2} } \right) + 0.1030198229{ }\left( {\tau^{*} } \right)^{4} \\ & \quad + 0.03238695124{ }\left( {\tau^{*} } \right)^{6} + 0.01234307481{ }\left( {\tau^{*} } \right)^{8} + \cdots \cdots \cdots. \\ \end{aligned}$$

## Discussion of results

**Figure 2:**
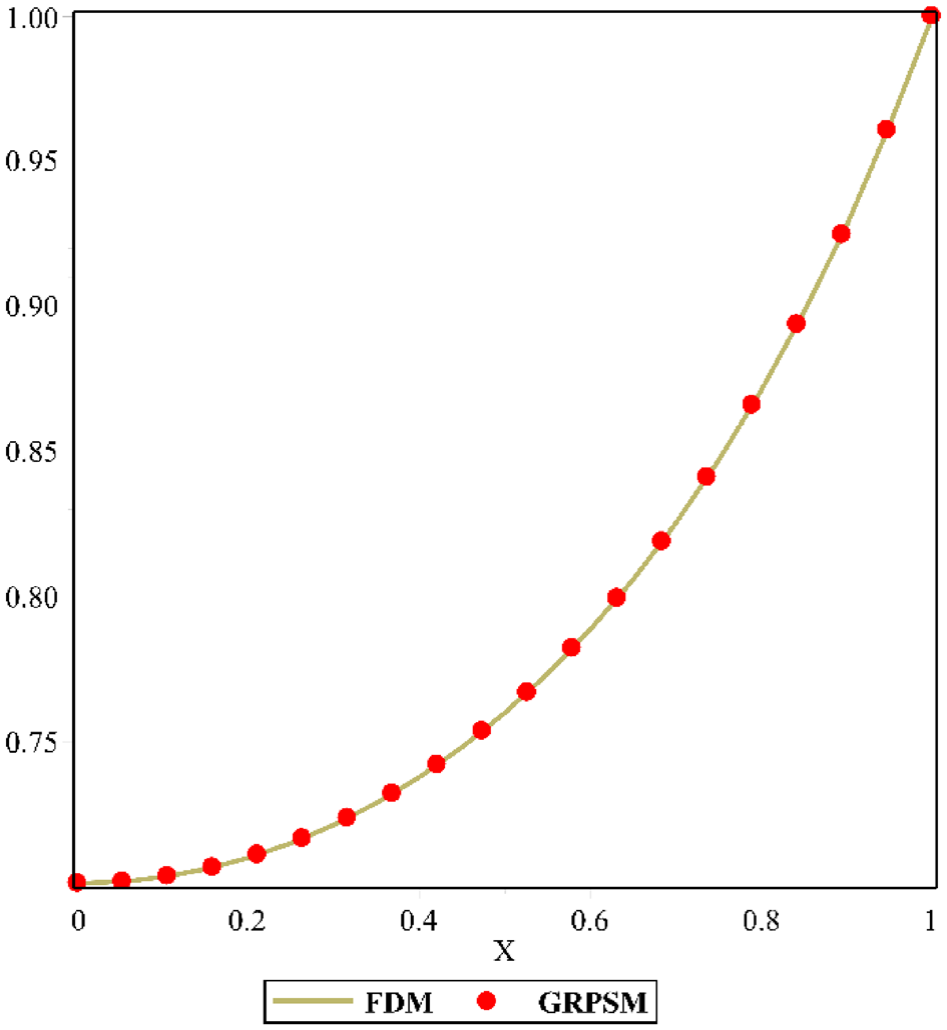
Validation of the present result with the numerical method.

**Figure 3:**
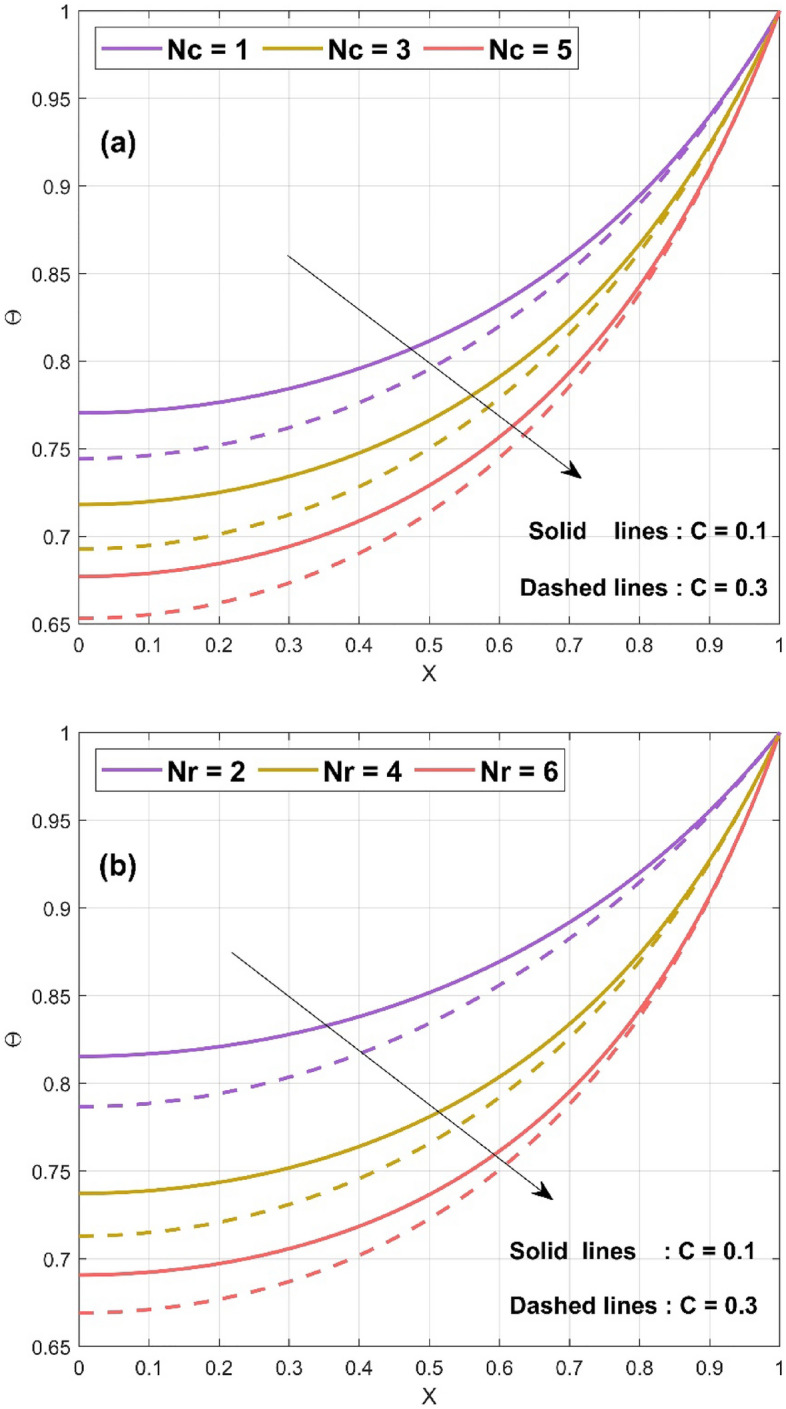
(**a**) Nature of $\Theta \left( {X,\tau^{*} } \right)$ for various $Nc$ values (**b**) Nature of $\Theta \left( {X,\tau^{*} } \right)$ for various $Nr$ values.

**Figure 4:**
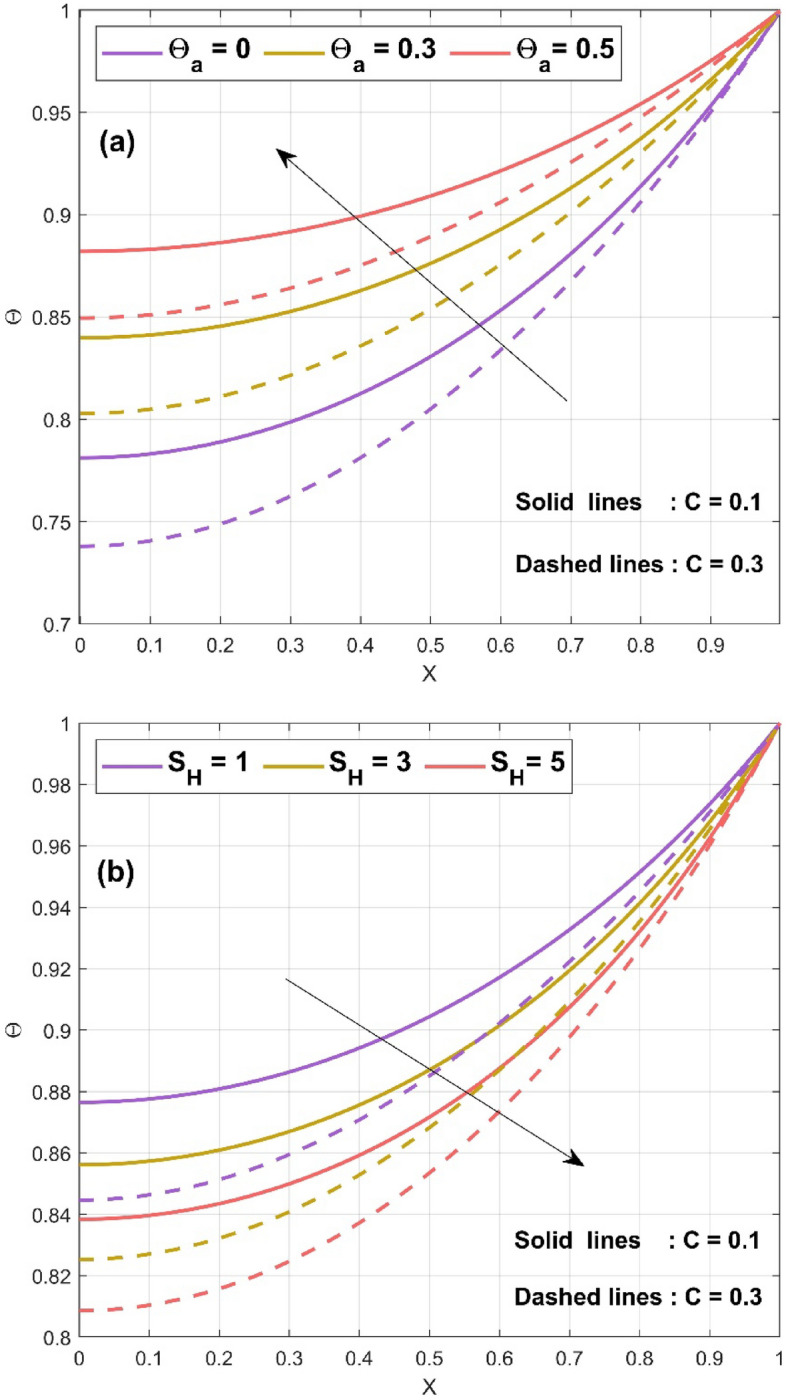
(**a**) Nature of $\Theta \left( {X,\tau^{*} } \right)$ for various $\Theta_{a}$ values (**b**) Nature of $\Theta \left( {X,\tau^{*} } \right)$ for various $S_{H}$ values.

**Figure 5:**
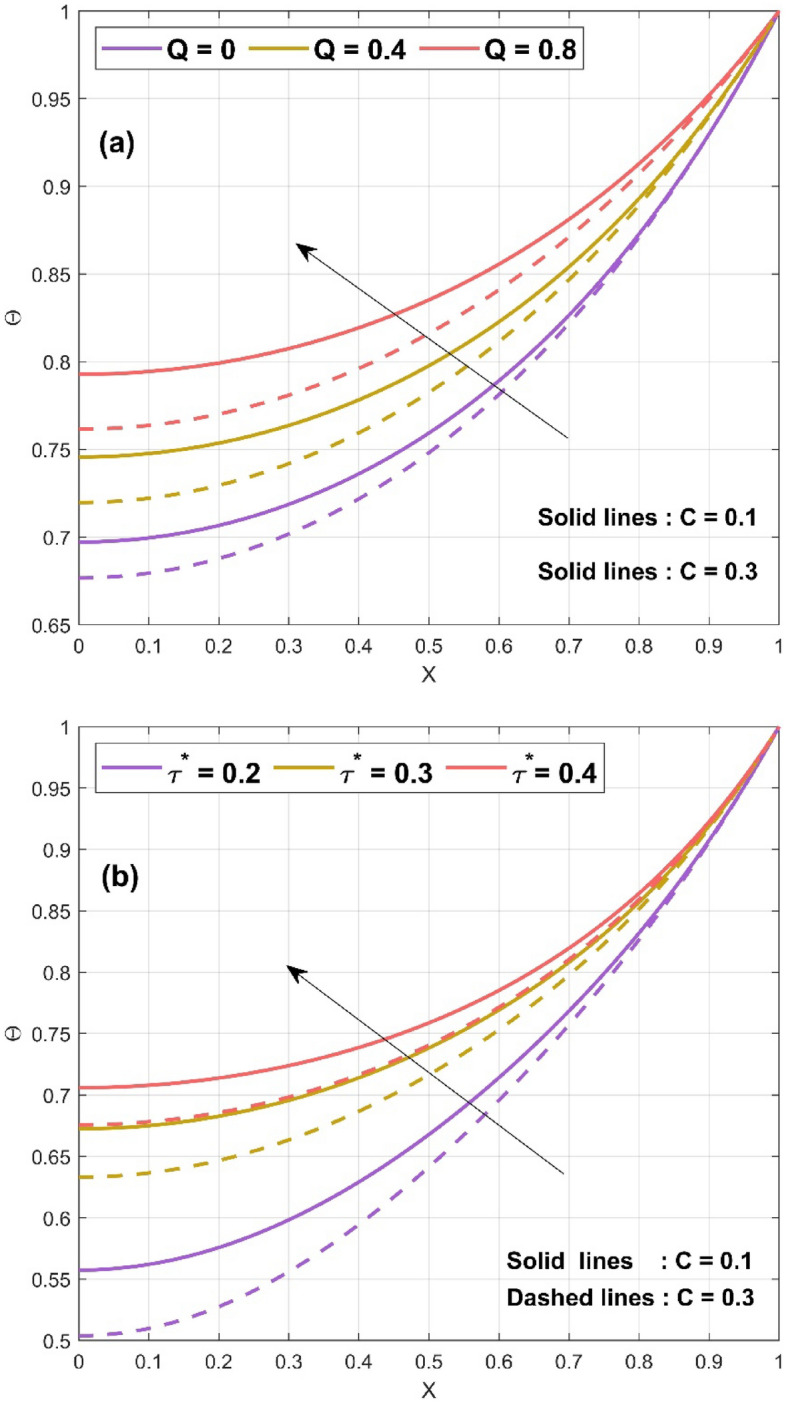
(**a**) Nature of $\Theta \left( {X,\tau^{*} } \right)$ for various $Q$ values (**b**) Nature of $\Theta \left( {X,\tau^{*} } \right)$ for various τ* values.

**Figure 6:**
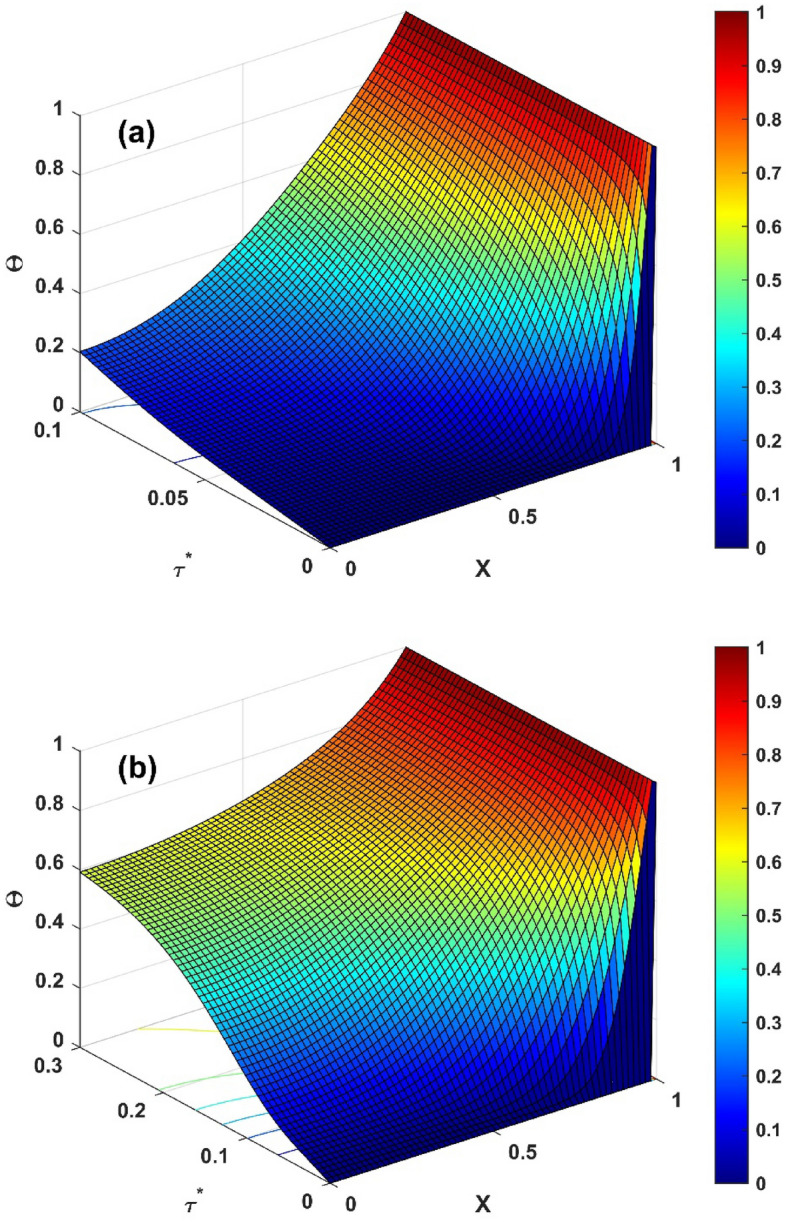
(**a**) Nature of $\Theta \left( {X,\tau^{*} } \right)$ for $\tau^{*} = 0.1$ (**b**) Nature of $\Theta \left( {X,\tau^{*} } \right)$ for various $\tau^{*} = 0.3$.

**Figure 7:**
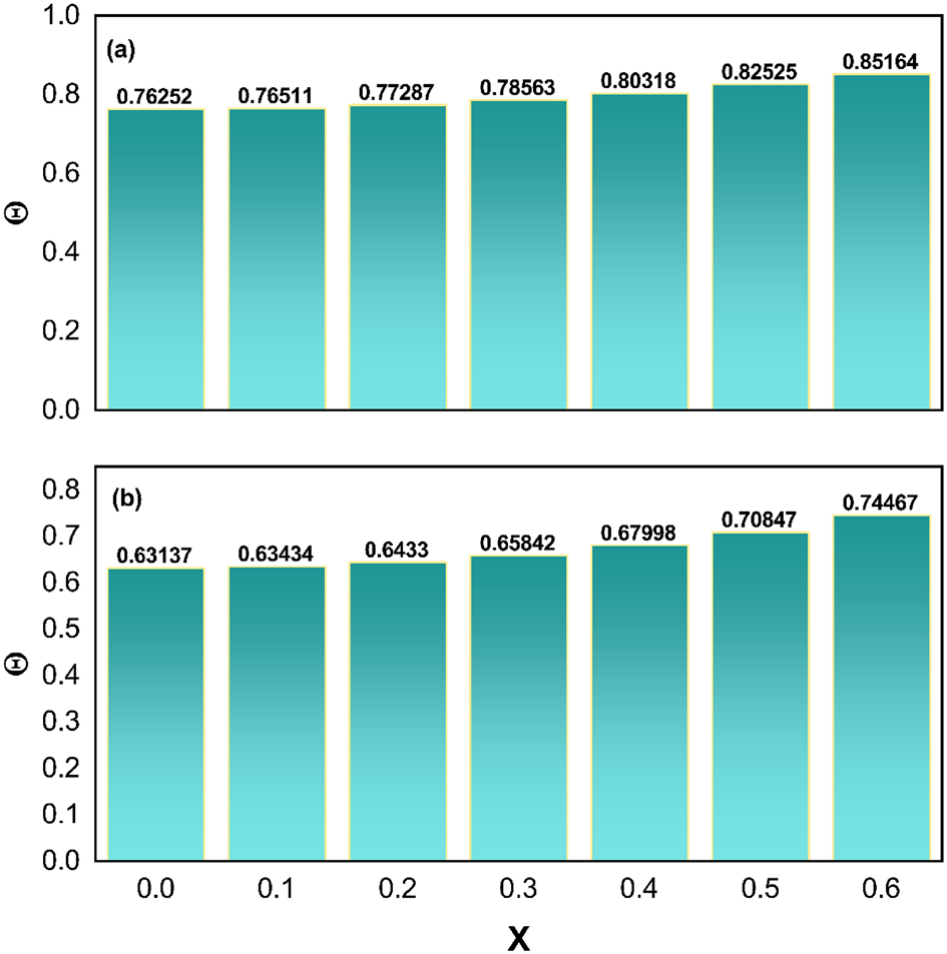
(**a**) Nature of $\Theta \left( {X,\tau^{*} } \right)$ of solid non-porous fin (**b**) Nature of $\Theta \left( {X,\tau^{*} } \right)$ of porous fin.

An internal heat generation, natural convection, and radiation impact are taken into account in formulating the unsteady thermal model of an inclined concave porous fin. Equation (1) expresses the corresponding balanced heat equation and is converted to a PDE using dimensionless terms along with BCs. The obtained equation discloses that dimensionless parameters effect the thermal distribution of a porous fin. Subsequently, Eq. (5) is derived analytically using the elementary properties of the proposed technique. For the analysis of the inclination effect, Table 1 is provided to witness the variation in the transient thermal profile $\Theta \left( {X,\tau^{*} } \right)$ of the inclined porous fin with respect to different angles of inclination $\alpha$. It is detected from this table, that thermal distribution diminishes from base to tip of the porous fin for all non-dimensional variables considered in the study $Nc = 1$, $S_{H} = 10$, $Nr = 1$, $\Theta_{a} = 0.1$, $k_{r} = 0.1$, $C = 0.1$, $\phi = 0.1$, $\gamma = 0.1$, $Q = 0.8$ at different values of inclination angle. The thermal profile signifies greater thermal distribution at $\alpha = 0$, resulting in a lower rate of heat transfer. Meanwhile, as the $\alpha$ value is modified ($\alpha = {\pi \mathord{\left/ {\vphantom {\pi 6}} \right. \kern-\nulldelimiterspace} 6},\,{\pi \mathord{\left/ {\vphantom {\pi 3}} \right. \kern-\nulldelimiterspace} 3},{\pi \mathord{\left/ {\vphantom {\pi 2}} \right. \kern-\nulldelimiterspace} 2}$), the thermal profile of the fin reveals decreasing characteristics at all locations of fin length. The consequence of the aforementioned dimensionless parameters on the thermal gradient $\Theta \left( {\tau^{*} ,X} \right)$ of the fin is graphically examined in this section. The nonlinear PDE (Eq. (5)) is solved using the finite difference method (FDM) technique in the domain $0 \le X \le L$ and $0 \le \tau^{*} \le T$. Along with uniform mesh, the finite-difference approximation is applied in the direction of $X$ and the step size of time and space domains are chosen as $\Delta \tau^{*} = \Delta X = 0.001$. Further, the present analysis (GRPSM) is compared with the numerical result (FDM) as displayed in Fig. 2 and they are found to be in excellent agreement. The significance of dimensionless parameters on the temperature deviance of the fin has been portrayed in Figs. 3, 4, 5, 6 and 7.

**Table 1:** Variation of $\Theta \left( {X,\tau^{*} } \right)$ for various angle of inclination at $\tau^{*} = 0.5$.

$X$	$\Theta \left( {X,\tau^{*} } \right)$			
	$\alpha = 0$	$\alpha = \frac{\pi }{6}$	$\alpha = \frac{\pi }{3}$	$\alpha = \frac{\pi }{2}$
0	0.762517721	0.746150111	0.734722554	0.730650835
0.1	0.765114321	0.748806389	0.737418358	0.733360298
0.3	0.785633513	0.769877478	0.758858153	0.754927991
0.5	0.825253915	0.810996009	0.800992971	0.797418843
0.7	0.882180478	0.871154813	0.863380424	0.860594648
0.9	0.956000595	0.951191296	0.94777482	0.946545365
1	1	1	1	1

The impact of convective parameter $Nc$ on the temperature distribution of the concave fin is seen in Fig. 3a for $S_{H} = 0.5$, $Nr = 1$, $\Theta_{a} = 0.1$, $k_{r} = 0.1$, $\phi = 0.1$, $\gamma = 0.1$, $\alpha = {\pi \mathord{\left/ {\vphantom {\pi 6}} \right. \kern-\nulldelimiterspace} 6}$, $Q = 0.8$, and $\tau^{*} = 0.5$ by considering different $C$ values ($C = 0.1\,{\text{and}}\,C = 0.3$). It is detected that with a rise in the convective parameter ($Nc = 1,\,3,\,5$) thermal profile decreases. This is due to the effect of the natural convection on the concave surface of the fin. The convection will carry the heat on fin surface and hence helps in decreasing the heat and aids in fin cooling. With all the considered parameter $Nc = 1$, $S_{H} = 0.6$, $\Theta_{a} = 0.2$, $k_{r} = 0.1$, $\phi = 0.1$, $\gamma = 0.1$, $\alpha = {\pi \mathord{\left/ {\vphantom {\pi 6}} \right. \kern-\nulldelimiterspace} 6}$, $Q = 0.8$, Fig. 3b displays the impact of radiative parameter $Nr$ on the thermal performance of the concave fin. The radiative parameter exhibits the same nature as that of the convective parameter, i.e., a decrease in temperature profile with increase in $Nr$ (2, 4, 6). The radiation effect increases the transfer of heat from fin surface to the surrounding. Therefore, the reduction in temperature has been observed with rising in radiation parameter. The significance of ambient temperature $\Theta_{a} \left( {0,\,0.3,\,0.5} \right)$ on the fin thermal field of a concave inclined porous fin with $Nc = 1$, $S_{H} = 0.2$, $Nr = 1$, $k_{r} = 0.1$, $\phi = 0.1$, $\gamma = 0.1$, $\alpha = {\pi \mathord{\left/ {\vphantom {\pi 6}} \right. \kern-\nulldelimiterspace} 6}$, $Q = 0.8$, and $\tau^{*} = 0.5$ is exhibited in Fig. 4a. The enhancement in ambient temperature results in an increase in thermal profile. This is because, $\Theta_{a}$ is the ratio of surrounding temperature to base temperature. Hence, with augment in $\Theta_{a}$ there is a decrease in the transmission of heat from fin surface to surrounding which leads to the increase in thermal profile. By setting $Nc = 2$, $Nr = 1$, $\Theta_{a} = 0.3$, $k_{r} = 0.1$, $\phi = 0.1$,$\gamma = 0.1$ , $\alpha = {\pi \mathord{\left/ {\vphantom {\pi 6}} \right. \kern-\nulldelimiterspace} 6}$, $Q = 0.7$, and $\tau^{*} = 0.5$, the effect of $S_{H}$ on $\Theta$ of the fin has been revealed in Fig. 4b. The enhancement in $S_{H}$ ($1,\,3,\,5$) leads to the reduction in the temperature profile. This is due to the fact that the porosity parameter helps in better interaction of surrounding air with the pores of the fin. Hence the porosity parameter helps in the fin cooling effect. The consequences of heat generation parameter $Q$ on the $\Theta$ of the fin have been depicted in Fig. 5a with $Nc = 1$, $S_{H} = 0.3$, $Nr = 1$, $\Theta_{a} = 0.2$, $k_{r} = 0.1$, $\phi = 0.1$, $\gamma = 0.1$, $\alpha = {\pi \mathord{\left/ {\vphantom {\pi 6}} \right. \kern-\nulldelimiterspace} 6}$, and $\tau^{*} = 0.5$. Here the augment in $Q$ ($0,\,0.4,\,0.8$) enlarges the thermal value along the axial length of the fin because of the presence of internal heat within the fin. The internal heat enhances the fin surface temperature therefore it decreases the rate of cooling from the surface of the fin. Figure 5b explicates the impact of $\tau^{*}$ on $\Theta$ of the concave porous fin. As the $\tau^{*}$ ($0.2,\,0.3,\,0.4$) values increase, the temperature improves remarkably under the consideration of $Nc = 1$, $S_{H} = 0.1$, $Nr = 1$, $\Theta_{a} = 0.1$, $k_{r} = 0.1$, $\phi = 0.1$, $\gamma = 0.1$, $\alpha = {\pi \mathord{\left/ {\vphantom {\pi 6}} \right. \kern-\nulldelimiterspace} 6}$, $Q = 0.8$. Figure 6a and b demonstrate the variation in the unsteady thermal distribution of the concave inclined porous fin as a function of time in a three-dimensional (3D) plot for $Nc = 1$, $S_{H} = 0.1$, $Nr = 1$, $\Theta_{a} = 0.1$, $k_{r} = 0.1$, $C = 0.3$, $\phi = 0.1$, $\gamma = 0.1$, $\alpha = {\pi \mathord{\left/ {\vphantom {\pi 6}} \right. \kern-\nulldelimiterspace} 6}$, and $Q = 0.8$. In particular, in Fig. 6a, the value of $\tau^{*}$ is picked as 0.1, and for this value, a decrement in thermal variation has been discovered, whereas an increment in thermal variation is recognized for $\tau^{*} = 0.3$, as exhibited in Fig. 6b. A comparison of the thermal profile values of the concave inclined solid and porous fin has been performed to uphold the significance of modeled fin problem. For a better interpretation of the thermal variation of the concave inclined porous and solid fin, Fig. 7a and b are plotted when the parameter values are set to $Nc = 1$, $Nr = 1$, $\Theta_{a} = 0.1$, $k_{r} = 0.1$, $C = 0.1$, $\gamma = 0.1$, $\alpha = {\pi \mathord{\left/ {\vphantom {\pi 6}} \right. \kern-\nulldelimiterspace} 6}$, $Q = 0.8$, and $\tau^{*} = 0.5$. At $X = 0$, the thermal field value of the solid fin $\left( {S_{H} = \phi = 0} \right)$ is comparatively higher than the porous fin $\left( {S_{H} = 10,\,\phi = 0.1} \right)$ and the same behavior is observed at all the considered values of $X$ (0, 0.1, 0.2, 0.3, 0.4, 0.5, 0.6). Thus, it is deduced that the porous fin helps in thermal dissipation and thereby provides a higher heat transfer rate. The effect of the fin taper ratio is recognizable in all of the aforementioned thermal investigation cases, and it is determined that the temperature inside the fin decreases as the scale of $C$ (0.1, 0.3) increases.

## Conclusions

The current study explains transient thermal dispersion in a concave permeable fin exposed to convective-radiative heat transfer. The governing equation is nondimensionalized by employing non-dimensional terms, and the arising PDE is solved analytically using the GRPSM. The consequences of significant non-dimensional factors on the temperature gradient are also depicted utilizing graphical portrayal. The following are some of the most significant study results of the present research:

- Unlike prior investigation, the heat transfer performance of the fin is effected not only by the radiation and convection mechanisms but also by the fin taper ratio, and inclination of the primary surface.
- The thermal dispersal in the porous fin is elevated in increasing time.
- With a greater level of fin taper ratio, the fin undergoes diminishing thermal dispersal.
- As the convection-conduction and porosity parameter values rise, the thermal dispersal in the fin declines.
- The temperature profile of the fin exhibits an enhanced nature for the raised heat-generating parameter.
- The thermal dispersal in the fin drops as the magnitude of the radiation-conduction attribute rises.
- It may be highly beneficial to a designer to select the tapered fin design with an inclined vertical surface for thermal effectiveness in a practical application such as latent heat thermal energy storage systems.
- GRPSM provides an analytical solution for nonlinear differential equations and the obtained outcomes in this research work are in close agreement with numerical ones. The exactness of the proposed method signifies that GRPSM is an alternative to other techniques for solving nonlinear PDEs.

## Data availability

All the data are clearly available in the manuscript.

## Abbreviations

$$k$$: Thermal conductivity
$Nc$: Convection–conduction parameter
$\alpha$: Angle of inclination
$$\tau$$: Time
$$\varepsilon^{*}$$: Emissivity
$\sigma$: Stefan–Boltzmann constant
$g$: Acceleration due to gravity
$$\Lambda$$: Fin taper
$$K$$: Permeability
$$c_{p}$$: Specific heat
$Nr$: Radiation–conduction parameter
$h^{*}$: Convective heat transfer coefficient
$$\rho$$: Density
$W$: Width of the fin
$\Theta$: Non-dimensional temperature
$t(x)$: Local semi-fin thicknesses
$$\phi$$: Porosity
$C$: Fin taper ratio
$t_{b}$: Semi-base thickness
$X$: Fin’s length (dimensionless)
$Q$: Internal heat generation parameter (dimensionless)
$$S_{H}$$: Porosity parameter
$L$: Length of the fin
$$\tau^{*}$$: Time(dimensionless)
$q^{*} (T)$: Internal heat generation
$\beta$: Volumetric expansion index
$x$: Fin axial distance
$$T$$: Temperature

## Subscripts

$$a$$: Ambient
$$b$$: Base
$$s$$: Solid
$$r$$: Relative quantity

## References

[1] Aly EH, Pop I (2020). MHD flow and heat transfer near stagnation point over a stretching/shrinking surface with partial slip and viscous dissipation: Hybrid nanofluid versus nanofluid. Powder Technol..

[2] Song Y-Q (2021). Bioconvection analysis for Sutterby nanofluid over an axially stretched cylinder with melting heat transfer and variable thermal features: A Marangoni and solutal model. Alex. Eng. J..

[3] Bilal M, Arshad H, Ramzan M, Shah Z, Kumam P (2021). Unsteady hybrid-nanofluid flow comprising ferrousoxide and CNTs through porous horizontal channel with dilating/squeezing walls. Sci. Rep..

[4] Madhukesh J (2021). Physical insights into the heat and mass transfer in Casson hybrid nanofluid flow induced by a Riga plate with thermophoretic particle deposition. Proc. Inst. Mech. Eng. Part E J. Process Mech. Eng..

[5] Waini I, Ishak A, Pop I, Nazar R (2021). Dusty hybrid nanofluid flow over a shrinking sheet with magnetic field effects. Int. J. Numer. Methods Heat Fluid Flow.

[6] Nadeem M, Siddique I, Awrejcewicz J, Bilal M (2022). Numerical analysis of a second-grade fuzzy hybrid nanofluid flow and heat transfer over a permeable stretching/shrinking sheet. Sci. Rep..

[7] Algehyne EA (2022). Investigation of thermal performance of Maxwell hybrid nanofluid boundary value problem in vertical porous surface via finite element approach. Sci. Rep..

[8] Ndlovu PL, Moitsheki RJ (2020). Steady state heat transfer analysis in a rectangular moving porous fin. Propuls. Power Res..

[9] Madhura KR, Kalpana G, Makinde OD (2020). Thermal performance of straight porous fin with variable thermal conductivity under magnetic field and radiation effects. Heat Transf..

[10] Nabati M, Jalalvand M, Taherifar S (2021). Sinc collocation approach through thermal analysis of porous fin with magnetic field. J. Therm. Anal. Calorim..

[11] Kundu B, Yook S-J (2021). An accurate approach for thermal analysis of porous longitudinal, spine and radial fins with all nonlinearity effects: Analytical and unified assessment. Appl. Math. Comput..

[12] Buonomo B, Cascetta F, Manca O, Sheremet M (2021). Heat transfer analysis of rectangular porous fins in local thermal non-equilibrium model. Appl. Therm. Eng..

[13] Varun Kumar RS, Sarris IE, Sowmya G, Madhukesh JK, Prasannakumara BC (2022). Effect of electromagnetic field on the thermal performance of longitudinal trapezoidal porous fin using DTM-Pade approximant. Heat Transf..

[14] Aziz A, Fang T (2010). Alternative solutions for longitudinal fins of rectangular, trapezoidal, and concave parabolic profiles. Energy Convers. Manag..

[15] Torabi M, Aziz A, Zhang K (2013). A comparative study of longitudinal fins of rectangular, trapezoidal and concave parabolic profiles with multiple nonlinearities. Energy.

[16] Kang HS (2021). Analysis of a concave parabolic fin with vertically cutting fin tip and variable fin base thickness. Int. J. Heat Mass Transf..

[17] Wang F (2022). LSM and DTM-Pade approximation for the combined impacts of convective and radiative heat transfer on an inclined porous longitudinal fin. Case Stud. Therm. Eng..

[18] Goud JS (2022). Role of ternary hybrid nanofluid in the thermal distribution of a dovetail fin with the internal generation of heat. Case Stud. Therm. Eng..

[19] Jagadeesha KC (2022). A physical depiction of a semi-spherical fin unsteady heat transfer and thermal analysis of a fully wetted convective-radiative semi-spherical fin. J. Indian Chem. Soc..

[20] Das, R. & Kundu, B. New forward and inverse solutions for wet fins generalized profiles with all nonlinear phenomena. *J. Heat Transf.* **143** (2020).

[21] Zeeshan, A., Arain, M. B., Bhatti, M. M., Alzahrani, F. & Beg, O. A. Radiative bioconvection nanofluid squeezing flow between rotating circular plates: Semi-numerical study with the DTM-Padé approach. Mod. Phys. Lett. B (2021).

[22] John Christopher A, Magesh N, Punith Gowda RJ, Naveen Kumar R, Varun Kumar RS (2021). Hybrid nanofluid flow over a stretched cylinder with the impact of homogeneous–heterogeneous reactions and Cattaneo-Christov heat flux: Series solution and numerical simulation. Heat Transf..

[23] El Ibrahimi M, Samaouali A (2021). Closed-form approximate solution for heat transfer analysis within functionally graded plate with temperature-dependent thermal conductivity. Compos. Struct..

[24] Sowmya, G., Varun Kumar, R. S., Alsulami, M. D. & Prasannakumara, B. C. Thermal stress and temperature distribution of an annular fin with variable temperature-dependent thermal properties and magnetic field using DTM-Pade approximant. *Waves Random Complex Media* 0, 1–29 (2022).

[25] Varun Kumar, R. S. et al. Exploration of transient heat transfer through a moving plate with exponentially temperature-dependent thermal properties. Waves Random Complex Media **0**, 1–19 (2022).

[26] Sun Y (2022). Investigation of transient coupled conduction and radiation heat transfer in the linearly anisotropic scattering cylindrical medium by spectral collocation method. Int. J. Therm. Sci..

[27] Weera W (2022). Convective-radiative thermal investigation of a porous dovetail fin using spectral collocation method. Ain Shams Eng. J..

[28] Biswal U, Chakraverty S, Ojha BK, Hussein AK (2021). Numerical simulation of magnetohydrodynamics nanofluid flow in a semi-porous channel with a new approach in the least square method. Int. Commun. Heat Mass Transf..

[29] Abu Arqub O, Abo-Hammour Z, Al-Badarneh R, Momani S (2013). A reliable analytical method for solving higher-order initial value problems. Discrete Dyn. Nat. Soc..

[30] Abu Arqub O, El-Ajou A, Bataineh AS, Hashim I (2013). A representation of the exact solution of generalized lane-emden equations using a new analytical method. Abstr. Appl. Anal..

[31] Syam MI (2017). Analytical Solution of the Fractional Fredholm Integrodifferential Equation Using the Fractional Residual Power Series Method. Complexity.

[32] Az-Zo’bi EA, Yıldırım A, AlZoubi WA (2019). The residual power series method for the one-dimensional unsteady flow of a van der Waals gas. Phys. Stat. Mech. Appl..

[33] Chu YM, Nazir U, Sohail M, Selim MM, Lee JR (2021). Enhancement in thermal energy and solute particles using hybrid nanoparticles by engaging activation energy and chemical reaction over a parabolic surface via finite element approach. Fractal Fract..

[34] Zhao TH, Castillo O, Jahanshahi H, Yusuf A, Alassafi MO, Alsaadi FE, Chu YM (2021). A fuzzy-based strategy to suppress the novel coronavirus (2019-NCOV) massive outbreak. Appl. Comput. Math.

[35] Nazeer M, Hussain F, Khan MI, Rehman AU, El-Zahar ER, Chu YM, Malik MY (2022). Theoretical study of MHD electro-osmotically flow of third-grade fluid in micro channel. Appl. Math. Comput..

[36] Chu YM, Shankaralingappa BM, Gireesha BJ, Alzahrani F, Khan MI, Khan SU (2022). Combined impact of cattaneo-christov double diffusion and radiative heat flux on bio-convective flow of maxwell liquid configured by a stretched nano-material surface. Appl. Math. Comput..

[37] Zhao TH, Khan MI, Chu YM (2021). Artificial neural networking (ANN) analysis for heat and entropy generation in flow of non-Newtonian fluid between two rotating disks. Math. Methods Appl. Sci..

[38] Khan NM, Chu YM, Khan MI, Kadry S, Qayyum S (2020). Modeling and dual solutions for magnetized mixed convective stagnation point flow of upper convected Maxwell fluid model with second-order velocity slip. Math. Methods Appl. Sci..

[39] Ma J, Sun Y, Li B (2017). Simulation of combined conductive, convective and radiative heat transfer in moving irregular porous fins by spectral element method. Int. J. Therm. Sci..

[40] Modanli M, Abdulazeez ST, Husien AM (2021). A residual power series method for solving pseudo hyperbolic partial differential equations with nonlocal conditions. Numer. Methods Partial Differ. Equ..

