# Author Correction: Assessment of thermal distribution through an inclined radiative-convective porous fin of concave profile using generalized residual power series method (GRPSM)

**DOI:** 10.1038/s41598-022-19035-5

**Published:** 2022-08-29

**Authors:** R. S. Varun Kumar, G. Sowmya, M. C. Jayaprakash, B. C. Prasannakumara, M. Ijaz Khan, Kamel Guedri, Poom Kumam, Kanokwan Sitthithakerngkiet, Ahmed M. Galal

**Affiliations:** 1 Department of Mathematics, Davangere University, Davangere, Karnataka 577002 India; 2 Department of Mathematics, M S Ramaiah Institute of Technology, Bangalore, Karnataka 560054 India; 3 Department of Information Technology, University of Technology and Applied Sciences, Al Mussanah, Sultanate of Oman; 4 Department of Mathematics and Statistics, Riphah International University, I-14, Islamabad, 44000 Pakistan; 5 Nonlinear Analysis and Applied Mathematics (NAAM)-Research Group, Department of Mathematics, Faculty of Sciences, King Abdulaziz University, P.O. Box 80203, Jeddah, 21589 Saudi Arabia; 6 Mechanical Engineering Department, College of Engineering and Islamic Architecture, Umm Al-Qura University, P.O. Box 5555, Makkah, 21955 Saudi Arabia; 7 Center of Excellence in Theoretical and Computational Science (TaCS-CoE), KMUTT Fixed Point Research Laboratory, Room SCL 802 Fixed Point Laboratory, Science Laboratory Building, Departments of Mathematics, Faculty of Science, King Mongkut’s University of Technology Thonburi (KMUTT), 126 Pracha-Uthit Road, Bang Mod, Thung Khru, Bangkok, 10140 Thailand; 8 Department of Medical Research, China Medical University Hospital, China Medical University, Taichung, 40402 Taiwan; 9 Intelligent and Nonlinear Dynamic Innovations Research Center, Department of Mathematics, Faculty of Applied Science, King Mongkut’s University of Technology North Bangkok (KMUTNB), 1518, Wongsawang, Bangsue, Bangkok, 10800 Thailand; 10 Mechanical Engineering Department, College of Engineering, Prince Sattam Bin Abdulaziz University, Wadi ad-Dawasir, 11991 Saudi Arabia; 11 Production Engineering and Mechanical Design Department, Faculty of Engineering, Mansoura University, P.O. 35516, Mansoura, Egypt

Correction to: *Scientific Reports* 10.1038/s41598-022-15396-z, published online 02 August 2022

The original version of this Article contained an error in the Grant Code stated in the Acknowledgements section.

“The authors acknowledge the financial support provided by the Center of Excellence in Theoretical and Computational Science (TaCS-CoE), KMUTT. This research was funded by National Science, Research and Innovation Fund (NSRF), and King Mongkut’s University of Technology North Bangkok with Contract no. KMUTNB-FF-65-24. The authors would like to thank the Deanship of Scientific Research at Umm Al-Qura University for supporting this work by Grant Code: 22UQU4331317DSR18.”

now reads:

“The authors acknowledge the financial support provided by the Center of Excellence in Theoretical and Computational Science (TaCS-CoE), KMUTT. This research was funded by National Science, Research and Innovation Fund (NSRF), and King Mongkut’s University of Technology North Bangkok with Contract no. KMUTNB-FF-65-24. The authors would like to thank the Deanship of Scientific Research at Umm Al-Qura University for supporting this work by Grant Code: 22UQU4331317DSR25.”

The original Article has been corrected.

